# Serological Diagnosis of Liver Metastasis in Patients with Breast Cancer

**DOI:** 10.3969/j.issn.2095-3941.2012.01.011

**Published:** 2012-03

**Authors:** Rui Cao, Li-ping Wang

**Affiliations:** Department of Integrated TCM and Western Medicine, Tianjin Medical University Cancer Institute and Hospital, Tianjin 30060, China

**Keywords:** breast neoplasms, liver, neoplasm metastasis, oxidoreductases, gamma-glutamyltransferase

## Abstract

**Objective:**

To diagnose and explore the serological diagnostic factors for liver metastasis in patients with breast cancer before symptoms occur.

**Methods:**

A total of 430 female in-patients with breast cancer of stages 0 to IIIC who came to Tianjin Medical University Cancer Institute and Hospital from January 2003 to January 2004 were studied and followed up until May 2011. Serum levels of biochemical markers for tumor and liver were measured at the time of diagnosis.

**Results:**

Liver metastasis was more likely to occur in patients with stage III cancer or c-erbB-2-positive expression. Alanine aminotransferase, aspartate aminotransferase, γ-glutamyltransferase (GGT), alkaline phosphatase, lactate dehydrogenase (LDH), and carbohydrate antigen 153 (CA153) levels were significantly higher in patients with liver metastasis than those without liver metastasis. Diagnostic indices of LDH, GGT, and CA153 were 174 U/L, 32 U/L, and 26.48 µg/L, respectively. The areas under the curves of LDH, GGT, and CEA were 0.795, 0.784, and 0.661, respectively, and sensitivities of parallel tests for LDH and CA153 and for GGT and CA153 were 88.6% and 85.7%, respectively. The specificity of serial tests for both pairs of enzymes was 97.7%.

**Conclusions:**

The sensitivity and specificity of combined tumor and biochemical markers could be used as indicators during screening for breast-liver metastasis.

## Introduction

Breast cancer is the most common malignancy and the second most lethal cancer type in women worldwide^[^^1^^]^. Approximately 50% of all breast cancer patients will develop distant metastasis ^[^^2^^]^, and more than half of all patients with metastatic breast cancer will have liver involvement at some point ^[^^3^^]^. Patients receiving chemotherapy have relatively good overall prognoses, with many surviving for a median of 13 months ^[^^4^^]^. Hepatic metastasis has been found in 55% to 75% of all autopsies performed on patients who died from breast cancer ^[^^5^^]^. Moreover, hepatic metastasis is the rate-limiting factor for patient survival ^[^^6^^]^. Thus, the early diagnosis of liver metastasis from breast cancer is helpful for timely treatment, which in turn, favors better prognosis.

Fine-needle aspiration cytology (FNAC) of hepatic lesions has become a popular diagnostic tool because it renders accurate findings ^[^^7^^]^. However, it has not been advocated as a screening test because of its high risk of complications. A review of the literature shows that tumor seeding after fine-needle biopsy of the hepatocellular carcinoma may be observed in 0.6% to 5.1% of all cases. The use of FNAC in abdominal tumors is fatal in 0.006%-0.031% of cases. Most deaths are due to liver tumor hemorrhage ^[^^8^^]^. Hemobilia due to a portobiliary fistula is also a complication of fine-needle liver biopsies ^[^^9^^]^. Imaging modalities, such as contrast-enhanced computed tomography (CT), magnetic resonance imaging (MRI), contrast-enhanced ultrasound, and positron emission tomography CT (PET-CT), may diagnose liver metastasis from breast cancer ^[^^10^^, ^^11^^]^. However, a final diagnosis of early liver metastases from breast cancer is difficult to make using these modalities because of the absence of typical symptoms or signs. Symptomatic liver diseases (e.g., hepatomegaly, jaundice, and ascites) are discovered much later and bring about worse prognoses ^[^^12^^]^. Serological examination is used to monitor metastatic disease during treatment, although its accuracy is not very high ^[^^13^^]^. CA153 was found to be elevated above normal in 75.9% of all patients at diagnosis of metastasis ^[^^12^^, ^^14^^]^. Liver function tests showed poor results in 92% of all patients at presentation, with gamma glutamyl transferase (GGT) and alkaline phosphatase (ALP) being the most commonly elevated enzymes. As well, 54% of all patients showed aspartate transaminase (AST) levels of more than twice the upper limit of normal ^[^^15^^]^. HER-2/neu (c-erbB-2) overexpression was shown to enhance the metastatic potential of breast cancer cells due to its association with more aggressive clinicopathologic factors ^[^^16^^]^. In fact, in most patients, high values of the above indicies are the first signs of relapse.

The purpose of the present study was to determine whether biochemical hepatic tests or other tumor markers can be used to predict liver metastasis in patients with breast cancer.

## Materials and Methods

### Patients

Four hundred and thirty female in-patients with breast cancer of stages 0 to III_C_ in Tianjin Medical University Cancer Institute and Hospital between January 2003 and January 2004 were studied and followed up until May 2011.Written informed consent was obtained from all patients. Of these patients, 76 were confirmed with liver metastasis. Pathological testing for all patients was performed to confirm breast cancer. Contrast-enhanced CT, MRI, PET-CT, or biopsy was performed to confirm liver metastasis. Patients with a history of liver disease and those who did not undergo contrast-enhanced CT or MRI were excluded from the study.

### Investigated indices

Blind tests were performed to determine total bilirubin (TB), direct bilirubin (DB), alanine aminotransferase (ALT), aspartate aminotransferase (AST), serum total protein (TP), globulin (GLOB), albumin (ALB), γ-glutamyltransferase (GGT), alkaline phosphatase (ALP), lactate dehydrogenase (LDH), and carbohydrate antigen 153 (CA153) levels. Liver biochemical tests were performed within one week after liver metastasis was diagnosed by contrast-enhanced CT, MRI, PET-CT, or biopsy. Immunohistochemical testing for all patients was performed to confirm the expression of estrogen receptor (ER), progesterone receptor (PR), and cerbB-2. Pathological testing for all patients was performed to confirm TNM classification.

### Statistical analysis

Differences in clinical characteristics between patients with and without liver metastasis were analyzed by two-independent-sample tests. One-sample Kolmogorov-Smirnov tests were used to determine the distribution of ALP, TP, ALB, GLOB, GGT, ALT, AST, TBIL, DBIL, LDH, and CA153. Data with skewed distributions were presented as medians (quartile interval). Two-independent-sample and χ^2^ tests were also used to determine significant differences between patients with and without liver metastasis. Cox regression analysis was performed on GGT, ALP, LDH, TB, DB, ALT, AST, TP, GLB, ALB, and CA153 findings to determine characteristic factors for survival time. Screening tests for LDH, GGT, and CA153, as well as parallel and serial tests for CA153 and LDH and for CA153 and GGT, were used to determine diagnostic factors for liver metastasis in patients with breast cancer. Statistical analysis was performed using SPSS. *P*<0.05 indicates a significant difference (version 16.0, Chicago, IL, USA).

## Results

### Patients’ characteristics

Patient ages ranged from 25 years to 86 years with a median of 50 years. No significant difference was found in age between patients with and without liver metastasis (*P*=0.212). TNM staging, lymph node metastasis, and expression of c-erbB-2 were significantly different between patients with and without liver metastasis. No significant difference was found between primary tumor status and the expression of ER and PR (**Table 1**). Liver metastasis rates gradually increased with increasing number of lymph node metastasis. In addition, liver metastasis was more likely to occur in patients with stage III cancer or cerbB 2-positive expression than those with cancers of stages I or II and those without c-erbB-2 expression.

**Table 1:** Patients’ characteristics.

	With liver metastasis	Without liver metastasis	Transfer rate (%)	Z	*P*
Primary tumor (UICC stage)					
T_0_	0	2	0.00	-0.59	0.555
T_1_	17	102	14.29		
T_2_	52	211	19.77		
T_3_	3	35	7.89		
T_4_	3	4	42.86		
Lymph node metastasis (UICC stage)					
N_0_	12	110	9.84	-3.883	<0.001
N_1_	20	112	15.15		
N_2_	12	65	15.58		
N_3_	31	67	31.63		
TNM staging (UICC stage)					
0	0	2	0.00	-2.961	0.003
I	6	43	12.24		
II	24	168	12.50		
III	45	141	24.19		
Immunohistochemical testing					
ER+	39	171	18.57	-0.347	0.729
ER-	45	178	20.18		
PR+	32	161	16.58	-0.466	0.641
PR-	44	188	18.97		
HER-2/neu (cerbB-2)+	38	109	25.85	-3.024	0.002
HER-2/neu (cerbB-2)-	34	233	12.73		

### Survival analysis and Cox regression

**Figure 1:**
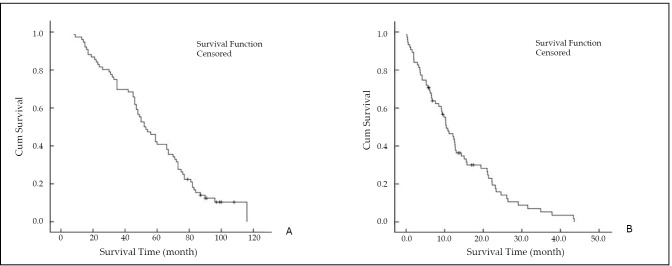
Survival analysis of breast cancer patients with liver metastasis. (A) Survival time of liver metastasis. (B) Survival time from liver metastasis to death. The median survival time was 54.3 months for patients with liver metastasis, and the median survival time from liver metastasis to death was 11.2 months.

The mean survival times of all patients, patients with liver metastasis, and patients without liver metastasis patients were (92.2±1.7) months, (57.1±3.5) months, and (89.4±1.3) months, respectively. The median survival time was 54.3 months for patients with liver metastasis, and the median survival time from liver metastasis to death was 11.2 months (**Figure 1**). Cox regression analysis showed that ALB and TB levels and primary tumor and lymph node metastases were significantly correlated with the survival time of breast cancer patients with liver metastasis (*P*<0.05, **Table 2**).

**Table 2:** Cox regression analysis of breast cancer patients with liver metastasis.

	**χ**^2^	*P*
Serum total protein (g/L)	0.219	0.640
Serum albumin (g/L)	9.917	0.002
Alanine aminotransferase (U/L)	0.454	0.501
Aspartate aminotransferase (U/L)	0.655	0.418
Total bilirubin (μmol/L)	7.210	0.007
Direct bilirubin (μmol/L)	0.241	0.624
γ-glutamyltransferase (U/L)	1.630	0.202
Alkaline phosphatase (U/L)	0.030	0.862
Lactate dehydrogenase (U/L)	0.134	0.714
Carbohydrate antigen 153 (μg/L)	0.002	0.181
Primary tumor (T)	13.671	<0.001
Lymph node metastasis (N)	4.699	0.034

### Test of normality and two-independent-sample tests

One-sample Kolmogorov-Smirnov test showed that the distributions of GLOB, ALB, ALB/GLOB, ALP, GGT, ALT, AST, TBIL, DBIL, LDH, and CA153 were skewed. GGT, ALT, AST, LDH, ALP, and CA153 levels were significantly higher in patients with liver metastasis than in those without liver metastasis (*P*<0.05, **Table 3**). Patients with c-erbB-2-positive expression had a higher risk of liver metastasis than those with c-erbB-2-negative expression (χ^2^=9.177, *P*=0.002). No significant difference was found in the ALB, GLOB, ALB/GLOB, TBIL, and DBIL levels between patients with and without liver metastasis (data not shown). No significant differences in the serological factors described above, except for CA153 (*P*<0.05), were found among patients with distant metastasis in other parts of the body, including the lung, brain and bone (data not shown).

**Table 3:** Comparison of serological factors.

	With liver metastasis	Without liver metastasis	*P*
LDH (U/L)	220 (224)	157 (43)	<0.001
ALT (U/L)	24 (34)	15 (10)	<0.001
AST (U/L)	30 (35)	17 (6.25)	<0.001
GGT (U/L)	41 (83)	19 (12.25)	<0.001
ALP (U/L)	85 (106.75)	63 (28)	0.016
ALB (g/L)	41.35 (12.7)	42.15 (5.7)	0.093
GLOB (g/L)	31.47 (9.524)	30.85 (7.3)	0.154
TB (μmol/L)	9.8 (7.8)	9.95 (5.75)	0.505
CA153 (μg/L)	27.725 (87.175)	11.18 (8.54)	<0.001

GGT, g-glutamyltransferase; ALT, alanine aminotransferase; AST, aspartate aminotransferase; LDH, lactate dehydrogenase; ALP, alkaline phosphatase; ALB, albumin; GLOB, globulin; TB, total bilirubin; CA153, carbohydrate antigen 153.

### Screening test

**Figure 2:**
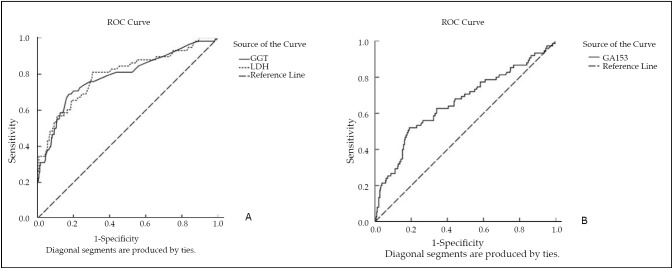
Receiver-operator characteristic curves of (A) LDH and GGT, and (B) CA153. The areas under the curves of LDH, GGT, and CEA were 0.795, 0.784, and 0.661, respectively (*P<*0.05). LDH, lactate dehydrogenase; GGT, g-glutamyltransferase; CA153, carbohydrate antigen 153.

Because the diagnostic indices of LDH, GGT, and CA153 at 174 U/L, 32 U/L and 24.17 µg/L, respectively, for screening liver metastasis were the greatest, cut-off points were selected as 174 U/L, 32 U/L, and 26.48 µg/L, respectively (**Figure 2**). The areas under the curves of LDH, GGT, and CA153 were 0.795, 0.784, and 0.661, respectively (*P*<0.05). The κ of parallel and serial tests for CA153 and LDH and for CA153 and GGT were 0.251 and 0.438, and 0.238 and 0.388, respectively. Other relevant indicators of screening tests are shown in **Table 4**.

**Table 4:** Screening tests of liver metastasis in patients with breast cancer.

	Sen (%)	Spe (%)	DI	CA	PV+	PV-	α	β	LR+	LR-
LDH	81.0	69.5	0.505	0.712	0.323	0.953	0.305	0.190	2.66	0.27
GGT	68.2	82.5	0.507	0.803	0.409	0.936	0.175	0.317	3.90	0.38
CA153	52.0	80.8	0.328	0.758	0.377	0.886	0.192	0.480	2.71	0.59
LDH and CA153 (serial test)	37.1	97.7	0.348	0.875	0.765	0.885	0.023	0.629	16.13	0.64
LDH and CA153 (parallel test)	88.6	56.9	0.455	0.622	0.292	0.961	0.431	0.114	2.06	0.20
GGT and CA153 (serial test)	32.4	97.7	0.301	0.871	0.733	0.905	0.023	0.676	14.09	0.69
GGT and CA153 (parallel test)	85.7	56.4	0.421	0.615	0.291	0.950	0.436	0.143	1.97	0.25

LDH, lactate dehydrogenase; GGT, g-glutamyltransferase; CA153, carbohydrate antigen 153; Sen, sensitivity; Spe, specificity; DI, diagnostic index; a, false positive rate; b, false negative rate; CA, crude accuracy; PV+, positive predictive value; PV-, negative predictive value; LR+, likelihood ratio positive; LR-, likelihood ratio negative.

## Discussion

In this study, analysis of the clinical characteristics of patients showed that the liver metastasis rate gradually increased with increasing number of lymph node metastasis. In addition, liver metastasis was more likely to occur in patients with stage III cancer or c-erbB-2-positive expression than in patients with cancer of stages I or II or c-erbB-2-negative expression ^[^^17^^, ^^18^^]^. Moreover, overexpression of c-erbB-2 was closely associated with increased angiogenesis and expression of vascular endothelial growth factor. Upregulation of vascular endothelial growth factor likely supports angiogenesis, promoting metastasis of tumor cells^[^^19^^]^.

Breast cancer has a clear tendency to spread to the lymph nodes, lungs, bones, and liver. Liver metastasis usually indicates the presence of disseminated cancer with very poor prognosis, even if it appears to be limited to a single organ ^[^^4^^]^. The mean survival time of all patients, patients with liver metastasis, and patients without liver metastasis were (92.2±1.7) months, (57.1±3.5) months, and (89.4±1.3) months, respectively. The median survival time was 54.3 months for patients with liver metastasis, and the median survival time from liver metastasis to death was only 11.2 months. Thus, liver metastasis results in significant reduction in average survival time.

Cox regression analysis confirmed that serum ALB and total bilirubin (TB) levels, which denote the presence of liver failure, are useful in predicting the duration of survival for patients with liver metastases, consistent with the findings of Wyld et al. ^[^^13^^]^. GGT, ALT, AST, ALP, LDH, and CA15-3 levels were also significantly higher in patients with liver metastasis than in those without liver metastasis.

Some studies have shown that CEA and CA153 are useful markers for the early diagnosis of metastases in patients with breast cancer ^[^^20^^-^^24^^]^. The sensitivity and specificity of combined CEA and LDH/GGT are adequate for screening colorectal liver metastasis ^[^^25^^]^. However, CA153 is more sensitive than CEA in patients with breast cancer ^[^^21^^]^. CA153 was highly associated with the number of positive lymph nodes and peritumoral lymphatic or blood vessel invasion ^[^^24^^]^. In fact, it is particularly valuable for monitoring distant metastasis that is absent of typical symptoms or signs and cannot be discovered early enough using existing radiological procedures ^[^^22^^]^. However, the low sensitivity of the tumor markers studied here limits their use in the clinical scale ^[^^23^^]^. Several other liver function tests have been used to evaluate hepatic function and screen for liver metastases ^[^^26^^-^^29^^]^.

A screening test was performed to determine whether LDH, GGT, and CA153 levels could be used to screen for liver metastasis. Because the diagnostic indices of LDH, GGT, and CA153 at 174 U/L, 32 U/L and 26.48 µg/L, respectively, for screening liver metastasis were the greatest, cut-off points were selected as 174 U/L, 32 U/L, and 26 µg/L, respectively. Sensitivity results of LDH, GGT, and CA153 yielded 81.0%, 68.2%, and 52.0%, respectively, while specificity results showed 69.5%, 82.5% and 80.8%, respectively. CA153 and GGT had high specificity and low sensitivity for diagnosing liver metastasis, whereas LDH had high sensitivity and low specificity for diagnosing liver metastasis.

The use of multiple screening tests for disease detection can improve the sensitivity or specificity of such screening tests. Thus, tumor markers in combination with serum liver function tests may improve the sensitivity and specificity of tests for screening liver metastases. In the present study, the sensitivity of parallel tests for LDH and CA153 and for GGT and CA153 were 88.6% and 85.7%, respectively. The specificity of serial tests for both pairs of tests was 97.7%. All examined parameters showed high negative predictive values (PV-), and only serial tests for LDH/CA 15-3 and GGTP/CA153 presented high positive predictive values (PV+) with high levels of OR. These results indicate that the sensitivity and specificity of tumor marker CA153 in combination with liver biochemical markers (LDH or GGT) are improved in patients with liver metastasis. Thus, combination of liver biochemical and tumor markers may be a good strategy for monitoring liver metastasis in breast cancer patients. When LDH>174 U/L and CA15-3>26 µg/L, or GGT>32 U/L and CA153>26 µg/L, contrast-enhanced CT, MRI, or PET-CT may be applied immediately to confirm liver metastasis. Timely treatment may improve the survival rate of breast cancer patients. This study shows that metastatic liver disease may be diagnosed pre-symptomatically and diagnosis of liver metastases can be more rapid and accurate.
